# Neonatal Survival Following Spontaneous Maternal Recovery From Ebola Virus Disease in a Resource-Limited Setting in Western Democratic Republic of the Congo

**DOI:** 10.1155/crdi/2987569

**Published:** 2025-03-14

**Authors:** Prince Imani-Musimwa, Emilie Grant, Daniel Mukadi-Bamuleka, Rigo Fraterne-Muhayangabo, Richard Kitenge-Omasumbu, Placide Mbala-Kingebeni, Zacharie Tsongo-Kibendelwa, Olivier Nyakio-Ngeleza, Ines Claris-Mwatsi, Juakali Sihali-Kyolov, Théophile Barhwamire-Kabesha, Celine Kavira-Malengera, Micheline Feza-Malira, Richard Bitwe-Mihanda, Dieudonné Sengeyi-Mushengezi-Amani, Mija Ververs

**Affiliations:** ^1^Department of Obstetrics and Gynecology, School of Medicine, University of Goma, Goma, Democratic Republic of the Congo; ^2^Département de Santé, Centre Régional d'Expertise en Recherche et Actions Humanitaires, Bukavu, Democratic Republic of the Congo; ^3^Department of Epidemiology, School of Public Health, University of Goma, Goma, Democratic Republic of the Congo; ^4^Département de Virologie, Institut National de Recherche Biomédicale (INRB), Kinshasa, Democratic Republic of the Congo; ^5^Department of Medical Biology, School of Medicine, University of Kinshasa, Kinshasa, Democratic Republic of the Congo; ^6^Department of Virology and Molecular Biology, Rodolphe Mérieux INRB-Goma Laboratory, Goma, North Kivu, Democratic Republic of the Congo; ^7^Centre des Opérations d'Urgences de Santé Publique (COUSP), Ministère de la Santé Publique, Hygiène et Prévention, Kinshasa, Democratic Republic of the Congo; ^8^Department of Internal Medicine, School of Medicine, University of Kisangani, Kisangani, Democratic Republic of the Congo; ^9^Department of Obstetrics and Gynecology, School of Medicine, Official University of Bukavu, Bukavu, Democratic Republic of the Congo; ^10^Department of Obstetrics and Gynecology, School of Medicine, University of Kisangani, Kisangani, Democratic Republic of the Congo; ^11^Department of Surgery, School of Medicine, Official University of Bukavu, Bukavu, Democratic Republic of the Congo; ^12^Département de Nutrition et santé, Centre de Recherche en Sciences Naturelles de Lwiro (CRSN-Lwiro), Bukavu, Democratic Republic of the Congo; ^13^Department of Pediatrics and Neonatology, School of Medicine, University of Goma, Goma, Democratic Republic of the Congo; ^14^Department of Obstetrics and Gynecology, School of Medicine, University of Kinshasa, Kinshasa, Democratic Republic of the Congo; ^15^Center for Humanitarian Health, Johns Hopkins Bloomberg School of Public Health, Baltimore, Maryland, USA

**Keywords:** DR Congo, Ebola and pregnancy, limited resources, neonatal survival, spontaneous recovery

## Abstract

**Background:** Pregnancy induces physiological decline in maternal immunity. Ebola virus disease (EVD) in pregnancy is associated with obstetrical complications, and vaccination in early pregnancy is recommended, but may not be without risk.

**Case Presentation:** We described neonatal survival after spontaneous maternal EVD recovery. This neonate was born to a 25-year-old mother admitted to an Ebola Treatment Unit (ETU) in July, 2020, after 11 days of symptoms. She was vaccinated with rVSV-ZEBOV three days before symptom onset and her real-time polymerase chain of reaction (RT-PCR) results confirmed EVD and malarial infection two days after, but she refused hospitalization. She was treated at home with PO ASAQ, amoxicillin, paracetamol, albendazole, omeprazole, and papaverine. Eleven days later, due to clinical deterioration and onset of vaginal hemorrhage, she finally accepted to be transferred in ETU. She was Parity 2, fetal age at admission was 5 weeks and 3 days. Upon admission, her EVD PCR measured NP 26.3 and GP 32.9. She did not receive monoclonal therapy against Ebola infection due to stock shortage. She received intravenous, artesunate, ceftriaxone, and papaverine. She experienced spontaneous resolution of EVD 18 days after symptom onset and was discharged. At 40 weeks gestation, seven and a half months after EVD recovery, she delivered a healthy female infant, APGAR 10/10/10, weighing 3450 g. Maternal blood, adnexal, and newborn blood samples were RT-PCR negative, and the mother and the baby were discharged after 14 days. At our last follow-up, in June 2023 (2 years, 3 months after delivery), the mother and the baby were in good health.

**Conclusion:** Neonatal survival following spontaneous maternal recovery from EVD in the first trimester of gestation is rare but possible, even in the context of limited clinical resources for treatment.

## 1. Background

Ebola virus disease (EVD) remains a deadly and highly contagious infection with a high case fatality rate (CFR) of 61% (95% confidence interval: 51.6–69.4) [1]. Ebola virus (EBOV) primarily spreads from a zoonotic reservoir to humans followed by human-to-human transmission, through bodily fluids and fomites [2]. Since 1976, the Democratic Republic of the Congo (DRC) has faced 15 outbreaks, all with the Ebola Zaire strain, except for the sixth outbreak [3]. The 11th Ebola outbreak (May–September 2020) occurred in the equatorial forest region with 130 cases including 55 deaths, representing a CFR of 42% [4]. Generally, there is no biological difference between men and women related to the transmission of or vulnerability to EBOV but women tend to bear a larger share of infections [5]. In pregnant women, who naturally experience a physiological decline in maternal immunity, EVD may lead to severe infection prompting several medical and obstetric complications (spontaneous abortion, premature rupture of the membranes (PRM), fetal death in utero (FDIU), perinatal hemorrhage, and maternal death) [6]. A review of 52 cases of pregnant women infected with EVD reported a maternal mortality rate of 72% in a sample size of 274 women and adverse pregnancy outcomes in 100% of the cases [7]. A study conducted during the 2014–2016 West Africa EVD outbreak reported a high risk of mortality in neonates born to mothers with EVD, and 83% of neonates in the study died [8]. A cohort study comparing the survival rate between pregnant versus nonpregnant women infected with EVD in DRC confirmed very poor fetal outcomes, regardless of maternal survival, with 41% of the pregnant EVD patients experiencing pregnancy loss [9]. Although vaccination against EVD induces maternal immunity with possibly no added risk of pregnancy loss [10, 11], the potential negative impact of vaccination side effects cannot be ruled out [11, 12]. Some cases of spontaneous maternal recovery from EVD have been documented; however, the neonatal survival rate remains very rare even in the case of monoclonal treatment during pregnancy [13–15]. In addition, data show after maternal recovery that the products of conception and breast milk may remain or become positive for Ebola viral RNA [7]. Here, we described the clinical course of a neonate born to a mother who spontaneously recovered from EVD in the first trimester of pregnancy after being treated in an ETU in the Equatorial region of DRC.

## 2. Case Presentation

A 25-year-old woman, weighing 78 kg, was admitted in July 2020 to an ETU in Equator Province during the 11th Ebola outbreak in DRC. Her clinical presentation included fever, generalized weakness, abdominal pain, and intense headache which began 10 days before. The patient was a direct contact case of her father, who died from his EVD five days before the onset of her symptoms. Due to her exposure, she was vaccinated with Ervebo (recombinant vesicular stomatitis virus—Ebola Zaire [rVSV-ZEBOV], Merck, Philadelphia, USA), three days before the onset of her symptoms.

Initially, she self-medicated with paracetamol and ibuprofen before being hospitalized in the ETU. Two days after symptom onset, she tested positive for EBOV using the GeneXpert assay (Cepheid, Sunnyvale, CA, USA) with cycle-threshold values (Ct-values) of nucleoprotein (NP) 22.4 and glycoprotein (GP) 27 indicating a high viral load (Table 1). She also tested positive for malaria (plasmodium falciparum) and pregnancy by rapid diagnostic tests (RDT). Despite confirmation of EBOV by RT-PCR and counseling, she did not consent to admission to the ETU, based on persistent rumors that EVD did not exist and that once in an ETU, patients would be inoculated with harmful products that could make them sicker and allow care providers to be funded. Thus, the medical team provided home consultation, set strict quarantine, and provided oral symptomatic treatment with artesunate–amodiaquine combination 100/270 mg twice daily for 3 days, amoxicillin 500 mg thrice daily for 10 days, paracetamol 1 g thrice daily for 5 days, albendazole 400 mg once daily, omeprazole 20 mg twice daily for 30 days, papaverine 40 mg thrice daily, folic acid 5 mg once daily for 30 days, and oral rehydration solution (ORS) to be taken as necessary. Her home treatment plan also included nutritional counseling and psychotherapy. Despite this home treatment regimen, the patient's clinical condition deteriorated, prompting her acceptance to transfer to the treatment center 10 days after symptom onset. Upon her admission at the ETU, she was Gravid 3, Parity 2, with her last menstrual cycle dating on 31st May, 2020, indicating gestational age of 5 weeks and 3 days. On admission, she was febrile (38.9°C), alert, and oriented x4. Her blood pressure was 120/80 mm Hg, heart rate 105 beats/minute, respiratory rate 26 breaths/minute, and arterial oxygen saturation (SaO_2_) at 98% on free air. The abdomen was soft with suprapubic tenderness. Gynecological and obstetrical examination showed a clean vulva with minimal bright redness; extra-uterine bleeding; posterior cervix, soft, long, firm, and closed; and uterus slightly increased in volume with sensitive adnexa.

Pregnancy and malaria tests were repeated and both remained positive. Hemoglobin was measured at 14 g/dL and blood glucose at 47 g/dL while urine dipstick showed leukocytosis. Her viral load had decreased on the repeat RT-PCR assay (Ct-values: NP 26.3 and GP 32.9) (Table 1). Without proper diagnostic or laboratory equipment in the ETU, we were unable to perform pelvic ultrasound, biochemistry tests to evaluate renal, liver or pancreatic function, or evaluate hemograms electrolyte panels or tests for inflammatory markers including C-reactive protein (CRP) levels.

Our diagnosis upon admission was infectious syndrome (EVD, malaria, and urinary tract infection) complicated by the threat of spontaneous abortion in evolutive pregnancy. We administered 50 mL bolus of glucose 50% followed by 500 mL of 10% Ringer's lactate solution to maintain hydration. Some oral medications, started at home (artesunate–amodiaquine combination, amoxicillin, paracetamol, and omeprazole), were stopped, and we continued intravenous with artesunate 180 mg twice daily for 4 days, ceftriaxone 2 g once daily for 7 days, metronidazole 500 mg once daily for 7 days, paracetamol 1 g thrice daily, tramadol 100 mg as needed, omeprazole 40 mg once daily, and papaverine 40 mg thrice daily. Per the randomized clinical controlled trial's extension protocol PALM (Pamoja Tulinde Maisha) [16], every confirmed EVD patient admitted in the ETU, was randomly allocated to receive one of the two available monoclonal antibody treatments: neutralizing monoclonal antibody (mAb114), Ebanga (Ansuvimab, Ridgepack Biotherapeutics, USA) or a neutralizing monoclonal, INMAZEB (REGN-EB3, Regeneron Pharmaceuticals). At the time of this patient's admission, the ETU had no stock of recommended Ebola antibody treatments, and therefore this patient never received monoclonal antibody treatment during her illness.

Five days after admission, we noted improved clinical and virological evolution, with RT-PCR results showing Ct-values of NP 33.4 and GP 38.5 (Table 1). On the 18th day from EVD diagnosis, 10th day of her ETU admission, and the 20th day from onset of symptoms, the patient's blood sample was tested negative for EBOV, and she was discharged five days later. One week postdischarge, we performed a pelvic ultrasound at a nearby health center that revealed an active pregnancy of 8 weeks and 2 days gestation.

After her discharge from the ETU, we conducted bimonthly follow-up visits to monitor the pregnancy which developed without complications, apart from a second malarial infection that was successfully treated during the second trimester.

The patient went into labor spontaneously at Week 40 of gestation (seven and a half months after Ebola recovery), and after 13 h , she gave birth to a female newborn by vaginal delivery in a health center. The infant's Appearance-Pulse-Grimace-Activity-Respiration (APGAR) scores were 10/10/10 at 1, 5, and 10 min after birth, respectively. All her reflexes were positive; she weighed 3450 g, with 35 cm head circumference and 51 cm length without apparent malformations. The newborn's blood test results showed a hemoglobin of 16 g/dL, blood glucose of 172 g/dL, and her SaO_2_ was 98% on free air. Maternal (breastmilk and blood) and fetal (blood, saliva, amniotic fluid, and placental blood) samples collected at birth were all negative for EBOV. The infant received supportive nutrition with intravenous fluids and ready-to-use infant formula milk while awaiting the RT-PCR results with a good digestive tolerance. Breastfeeding was commenced at Day 4 upon the reception of Ebola RT-PCR negative results for all samples tested. After 14 days, the mother and the infant were discharged from the maternity ward. The pair was followed in a health center weekly during the first month, and bimonthly until six months. We noted normal development of the infant's psychomotor and weight-for-stature during the follow-up period. At our last visit in mid-2023, the child was healthy at two years and three months old.

## 3. Discussion

This case study demonstrates spontaneous recovery from EVD and associated malarial and urinary tract coinfections in a pregnant woman infected during the first trimester of pregnancy and an uncomplicated fetal survival. Limitations of our study are mostly due to the unavailability of adequate laboratory and diagnostic equipment (for biochemistry results, hemograms, tests for inflammatory markers, and pelvic ultrasound) at the point of care during her illness, as well as absence of EBOV sequencing in the mother and virology and serology follow-up in the newborn. The missing clinical and diagnostic resources would have enabled more thorough surveillance and clinical case management. In utero, fetal susceptibility to pathogens corresponds with gestational age, and early pregnancy is the most vulnerable period. The synthesis of protective fetal antibodies (IgM and IgG) starts around the 20th week of gestation, and maternal antibodies (IgG) start crossing the placenta barrier from 13th weeks of gestational [17]. In addition, the synthesis of maternal anti-EVD immunoglobulins begins only after the sixth day symptom onset [18]. As their passage is impossible for fetal protection before 13 weeks, and our patient was infected at 5 weeks gestation, this child may not have received maternal IgG protection against EVD in utero [17, 18] during illness. Considering these factors, the immunological environment of maternal EVD in the first trimester of the pregnancy could have created conditions for spontaneous abortion; but this pregnancy persisted. High Ebola viral load is described in both the placenta and amniotic fluid in most cases of maternal EVD, which can cause FDIU even after maternal recovery [19, 20]. In this case study, fetal vulnerability was compounded further by maternal malarial infection, absence of monoclonal treatment, and the potential side effects of vaccination against EBOV as listed previously, which our patient received in the third week of her pregnancy. Moderate vaginal bleeding observed for three days upon her admission to the ETU could have been due to partial placental abruption. Without ultrasound equipment, we could not confirm this, although clinically partial abruption is the only plausible diagnosis. If further placental abruption occurred, consequences would have included not only destruction of the haematoplacental barrier, causing mixing of maternal and fetal blood and increased risk of a direct fetal EVD in utero, but also an increased risk of spontaneous abortion. Despite cumulative threats to both mother and fetus, all of which elevated the risk of spontaneous abortion, this pregnancy developed well resulting in a healthy newborn. Since most pregnancies that survive maternal EVD have generally a negative outcome [9, 21], the successful development and birth of an Ebola-free infant after spontaneous maternal recovery may challenge the theory of systematic vertical transmission of Ebola during pregnancy. A recent case report strengthens this hypothesis as it described the survival of a premature newborn without Ebola born to a mother in the acute phase of EVD [15]. In addition, we thought that if our patient had benefited the monoclonal therapy, she would early negativize her viral load. Therefore, during Ebola outbreak, availability of stocks of monoclonal therapy is essential in ETUs. In this case, the favorable clinical progress of the mother raises the question of whether EBOV strains of different virulence could have circulated during the 11th epidemic in DRC. Less virulent strains could have elicited maternal immunity without causing severe morbidity or mortality. It is also possible that the virus gradually lost its virulence through multiple passages in humans or due to the recurring nature of Ebola epidemics in this area causing host adaptation to the virus. A study carried out on 130 cases recorded in the 11th outbreak generated 87 genomes with two variants including Mbandaka in 84 (97%) cases and the Tumba variant in four cases. The Tumba variant exhibited a reduced substitution rate, suggesting transmission from a previous EVD survivor of ninth EVD outbreak in the same province [22]. However, Zaire strain was the sole strain in this outbreak, and severe EVD cases were still observed throughout this outbreak. In addition, the CFR during this outbreak was 42% which prompts us to look for additional causes, beyond the low virulence of the virus [4]. Furthermore, with such a trend of viral load, there is a possibility that detected particles of RNA by RT-PCR may no longer be able to fully replicate and that this may have had a positive impact on the infant status even though the mother displayed recognizable Ct values. We hypothesize that spontaneous maternal recovery from an EVD and associated fetal survival in this case study could have been linked to the absence of severe maternal comorbidities during the acute phase of the infection apart from malaria infection, vaccine-induced maternal immunity [11], and the supportive treatment our patient received upon diagnosis at home and during her admission in the ETU. Considering all the risk factors associated with this pregnancy, its development and subsequent neonatal survival are exceptional.

## 4. Conclusion

Spontaneous recovery from EVD and fetal survival during pregnancy in the first trimester remain rare but possible, even in limited resource settings, especially when specific therapies are unavailable. The efficacy of vaccination against EVD in early pregnancy to prevent maternal and/or fetal death should be further studied. The integration of and equipment in a maternity and neonatology service in the ETUs are essential for better management of neonates and obstetric patients.

## Figures and Tables

**Table 1:** Viral load evolution in spontaneous maternal recovery from Ebola infection.

Months	June							July															
Day	−3	−2	−1	1	2	3	4	5	6	7	8	9	10	11	12	13	14	15	16	17	18	19	20
Evenements	Vaccination			Symptom onset		Start PCR testing								Admission ETU									EBOV negative

Ct values (RT-PCR)	NP					22.4	22.9	23.4	23.9	24.1	24.8	25.3	25.9	26.3	27.3	27.5	29.1	33.4	35.3	36.3	37.4	39.8	40.9
	GP					27.9	28.2	28.7	29.0	29.5	29.9	30.7	31.0	32.9	33.9	35.0	36.7	38.5	38.9	39.1	39.7	40.8	42.5

Location and treatment	In community without treatment					In community with oral symptomatic treatment								In ETU with intravenous symptomatic treatment									

## Data Availability

The datasets used and/or analyzed for the current study are available from the corresponding author upon request.
